# Preparation and Characterization of Zinc(II)-Based Lewis/Brønsted Acidic Deep Eutectic Solvents

**DOI:** 10.3390/molecules28248054

**Published:** 2023-12-12

**Authors:** Chiara Pelosi, Aldo Quaranta, Marco Rollo, Elisa Martinelli, Celia Duce, Gianluca Ciancaleoni, Luca Bernazzani

**Affiliations:** 1 Department of Chemistry and Industrial Chemistry, University of Pisa, Via Giuseppe Moruzzi 13, 56124 Pisa, Italy; chiara.pelosi@unipi.it (C.P.); a.quaranta5@studenti.unipi.it (A.Q.); marco.rollo@phd.unipi.it (M.R.); elisa.martinelli@unipi.it (E.M.); celia.duce@unipi.it (C.D.); 2 Istituto Nazionale di Ottica (INO-CNR) Area di Pisa, Centro Nazionale delle Ricerche, Via Moruzzi, 56124 Pisa, Italy

**Keywords:** Lewis/Brønsted acidic deep eutectic solvents, LBDES, zinc (II), physicochemical properties of DESs

## Abstract

Lewis/Brønsted acidic deep eutectic solvents (LBDESs) are a recent class of solvents that combine the two types of acidity. In some cases, this synergy leads to enhanced catalytic properties for many reactions and applications. For this reason, it is important to discover more LBDESs. In this work, we prepared and characterized four different zinc(II)-based LBDESs, mixing ZnCl_2_ and various Brønsted acids: acetic, glycolic, levulinic, and formic acids. Apart from the latter, for which the corresponding DES is not thermally stable, the samples have been characterized in terms of density, viscosity, and conductivity. Notably, as zinc(II) is a diamagnetic metal, all of them are suitable for NMR spectroscopy, for example, for kinetic and mechanistic studies.

## 1. Introduction

Zinc(II) is acidic in nature, as the 4s and 4p orbitals are empty and available for coordination [1], whereas the 3d orbitals are full and, unlike many other late transition metals, redox-stable. This makes zinc(II) salts good catalysts for many reactions [2]: among others, we can remember the Friedel–Craft benzylation of benzenes [3], the Biginelli reaction [4], alkyne hydrogenation to alkenes [5], activation of CO_2_ [6], trans-amidation [7], and trans-esterification [8]. The latter has been also extended to polyester synthesis and degradation [9,10,11].

In the last few years, zinc(II) salts (generally chloride salts) have been used also for the preparation of acidic deep eutectic solvents (aDESs) [12]. In general, a DES is a mixture of hydrogen bond donors and acceptors (HBDs and HBAs, respectively) whose melting point is much lower than the value expected for an ideal mixture [13]. They recently found application in various fields, such as organic synthesis [14,15,16], metal deposition [17], extractive desulfurization [18], and CO_2_ capture [19,20]. DESs are usually prepared by heating (with conventional heating or microwave reactors [21]) the mixture of the precursors until a homogeneous liquid mixture is obtained, or in some cases by freeze-drying or evaporating the solvent in which the desired components are dissolved [22]. Their physicochemical and thermal properties have been extensively studied in view of orienting future applications [23,24,25,26]. In particular, the use of Zn(II) salts (generally chloride) as DES precursors enabled the preparation of new solvents for (i) cyclic voltammetry (with choline chloride [27]), (ii) electrolytes in zinc aqueous batteries (with lactic acid [28]), (iii) the fractionation of lignin (with acetamide [29]), (iv) cellulose dissolution (with water and formic acid [30]), and (v) the depolymerization of polyethylene terephthalate (with urea [31]) and polyethylene (with lactic acid [32,33]). Notably, the combination of ZnCl_2_ and acetic acid was known and characterized in 1939 [34] and used in different contexts [35,36]. This mixture, in fact, has peculiar properties and presents advantages with respect to the common use of the catalyst Zn acetate, as Zn acetate lacks any Brønsted acidity, and the presence of chloride ions of ZnCl_2_ could influence the solubility of many chemical species through the instauration of chemical equilibria or weak interactions.

In view of all these applications, the preparation of new Zn(II)-based DESs and their in-depth characterization proves to be a topic of recognized interest. This class of DESs presents the additional advantage of the possible synergy between Lewis and Brønsted acids (LBDES), which has been already assessed in a few cases [28,33,37,38,39,40,41,42,43], and more potential applications will be explored in the future. As is evident from some of these references, in some cases, the zinc-based solvent is catalytically active, meaning that one of the components of the DES directly enters into the mechanism of the reaction, lowering its activation barrier. Furthermore, unlike paramagnetic LBDESs based on iron, cobalt, or nickel, in the case of Zn(II)-based DESs, NMR spectroscopy can be used, for example, to study the kinetics of a reaction or better characterize the solute–solvent interactions, as has already been done for metal-free DESs [44].

For all these reasons, we report here the preparation and characterization of four LBDESs, based on zinc(II) and various Brønsted acids. Other diamagnetic metal salts have been tested, such as aluminum(III), zirconium(IV), and tin(II), but with these metals, we could not obtain a homogeneous liquid mixture. For the stable LBDESs (i.e., the ones formed using acetic, glycolic, and levulinic acids with ZnCl_2_), in-depth physicochemical characterization is provided in terms of viscosity, conductivity, and density. It is worth noting that the DES preparation was performed without the elimination of water traces from the precursors, to reproduce the conditions required for their application. In fact, for interesting applications, such as PET [37,42] or cellulose [45] hydrolysis, at least a small amount of water has to be present in the system. Overall, this study represents a necessary starting point for future applications of these systems in different fields.

## 2. Results and Discussion

### 2.1. DES Preparation and Stability

Given the versatility of iron(III)-based LBDESs [37], we confidently began mixing *d*^0^ and *d*^10^ transition metal salts with different carboxylic and sulfonic acids. Beyond the diamagnetic properties, all the metal salts we used are known for forming eutectic mixtures. For example, ZrOCl_2_·8H_2_O is a known precursor for acidic DESs [12], and generally, its acidity is due to the protons of water coordinated to the zirconium (Brønsted-type), rather than directly to the metal (Lewis-type). Regarding the organic acid, we tested a wide variety of substances, varying not only the strength, but also the presence of additional functional groups. In addition to this, only widespread and cheap acids have been tested, given that a solvent cannot be made of expensive components if practical applications are desired. Unfortunately, only a few combinations successfully led to the formation of a clear, homogeneous liquid, without an evident pattern. Table 1 shows the (few) LBDESs successfully prepared and the (many) unsuccessful combinations.

Some LA/LB mixtures, such as ZrOCl_2_·8H_2_O/MSA or SnCl_2_/glycolic acids, became unstable with time, meaning that after some hours they became heterogeneous again. For practical applications, this may not be a problem, especially if heating is required, but the characterization is difficult because the liquid tends to form a solid phase during the measurements. For these reasons, only zinc(II)-based LBDESs, which were stable for 24 h or longer, have been characterized here.

Prior to performing the evaluation of the samples’ properties, we estimated the amount of water present in each sample by performing Karl Fischer titrations and/or thermogravimetric analysis on the precursors. It is worth noting that water traces were not eliminated a priori from the precursors, to reproduce the conditions required for their application. Considering the water content of the single components (see Section 3), DES1–4 have approximately the following water content: 12,000, 30,000, 20,000, and 16,000 ppm, respectively, corresponding to 1.2%, 3%, 2%, and 1.6% of the total sample mass. More details on the calculation of water content are reported in Section 3, but it is worth remembering that the precise water content can vary from sample to sample, as it depends on exact reaction conditions and air exposure.

In addition, we noticed that DES4 showed instability during heating with gas evolution; therefore, in this case, the full characterization was unfeasible. In order to determine the nature of the evolved gas, two reactions have been hypothesized:

**Hypothesis** **1.** *ZnCl*_2_ *+ HCOOH* *⇌ ZnCl(HCOO) + HCl↑*

**Hypothesis** **2.** *ZnCl*_2_ *+ HCOOH* *⇌ ZnCl*_2_ *(H*_2_ *O) + CO↑*

During the heating, the evolved gas was collected, passed through a glass tube, and bubbled in an aqueous solution of AgNO_3_ (100 g/L), but no AgCl formed. In a different experiment, the glass tube was filled with CaCl_2_, to dry the gas, and then I_2_O_5_. The immediate formation of a purple phase (I_2_) upon gas/I_2_O_5_ contact (Figure S1) revealed the presence of a reducing gas, which confirmed hypothesis 2. This is coherent with the known dehydration of formic acid upon contact with drying agents such as H_2_SO_4_ or P_2_O_5_. In our case, “anhydrous” ZnCl_2_ acted as a drying agent. The release of CO makes the use of this DES unpractical for security reasons.

### 2.2. Melting Point

**Figure 1:**
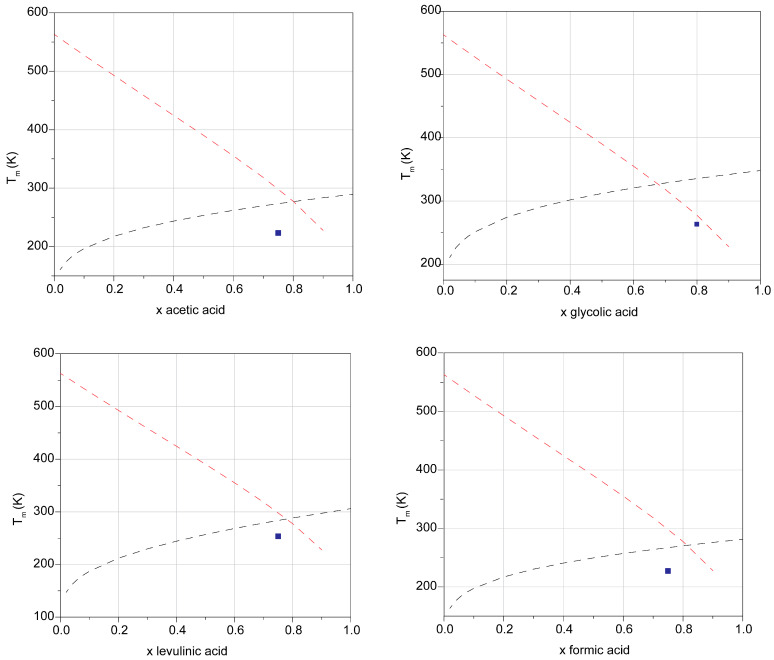
Experimental/theoretical solid–liquid phase diagrams for DES1–4. The dashed lines (black and red) are the ideal solubility curves of the solid–liquid phase diagrams of a binary mixture calculated by Equation (1), while the blue squares are the experimental melting temperatures obtained at a given molar ratio.

In order to verify whether the liquid phases produced by the interaction of ZnCl_2_ with Brønsted acids can be called DESs or not, the experimental melting points should be compared with the theoretical curves predicted for an ideal mixture (Equation (1)). The experimental melting points for DES1–4 were found to be −50, −10, −20, and −46 °C, respectively. It is useful to remember that anhydrous ZnCl_2_ melts at 290 °C. The comparison with ideal eutectic curves can be appreciated in Figure 1. 

In all the cases the theoretical curves cross each other between 0.6 and 0.8 (molar fraction of the Brønsted acid), in agreement with the experimental composition of the DESs (0.75 for DES1, DES3, and DES4; 0.8 for DES2). The activity coefficients are shown in Table 2. All activity coefficients found experimentally are obviously <1, a necessary condition for the system to be classified as a DES.

### 2.3. Density Measurements

**Figure 2:**
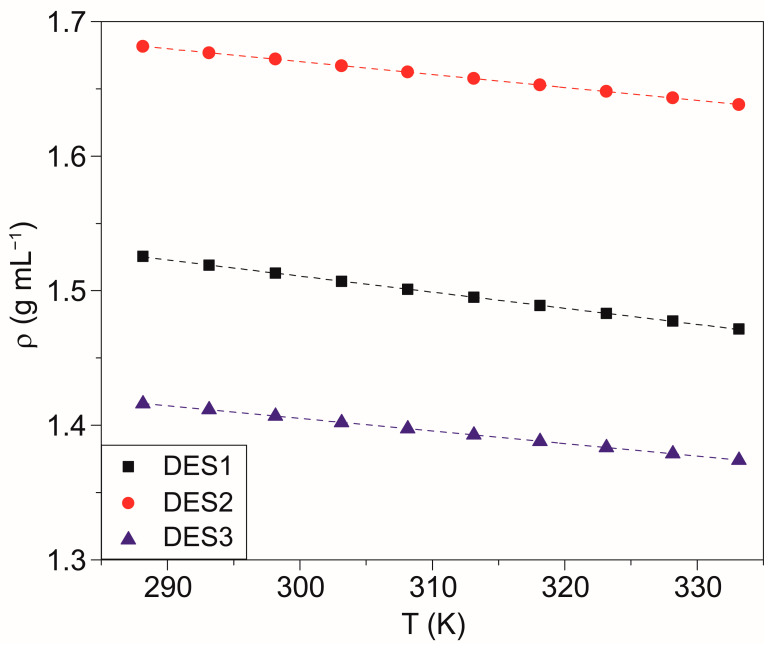
Density of samples measured in the temperature range 288.15–333.15 K. The dashed lines represent the linear fitting, achieved with an R^2^ > 0.9999.

The density of DES1–3 was measured in the 288.15–333.15 K range, giving the results summarized in Figure 2. The density of DES4 is not reported because the gas evolution with heating (resulting in the presence of numerous bubbles in the densimeter tube) and the sample chemical instability did not allow the recording of reliable measurements. The determination of the other physical properties (viscosity and conductivity) of this sample was hampered for the same reason.

The density values of DES1–3 are quite high when compared to those of organic DESs [25,26], but they are comparable to those of other DESs containing metal salts [13,22,38]. Furthermore, it is interesting to note that the values are higher than those of the respective acid precursors (density at 298.15 K of acetic acid: 1.05 g·cm^−3^; glycolic acid: 1.490 g·cm^−3^; levulinic acid: 1.136–1.147 g·cm^−3^) [46], suggesting that the addition of ZnCl_2_ leads to an increase in the average packing of the systems. The density dependency on temperature was described with a linear fitting (R^2^ > 0.9999), which was used in turn to calculate the coefficient of isobaric expansibility (*α_P_*), reported in Table 3. *α_P_* describes the system’s capability to expand when heated in isobaric conditions. The values obtained are higher than those reported for other organic DESs (e.g., choline chloride with organic acids) [25,26].

### 2.4. Conductivity Measurements

**Figure 3:**
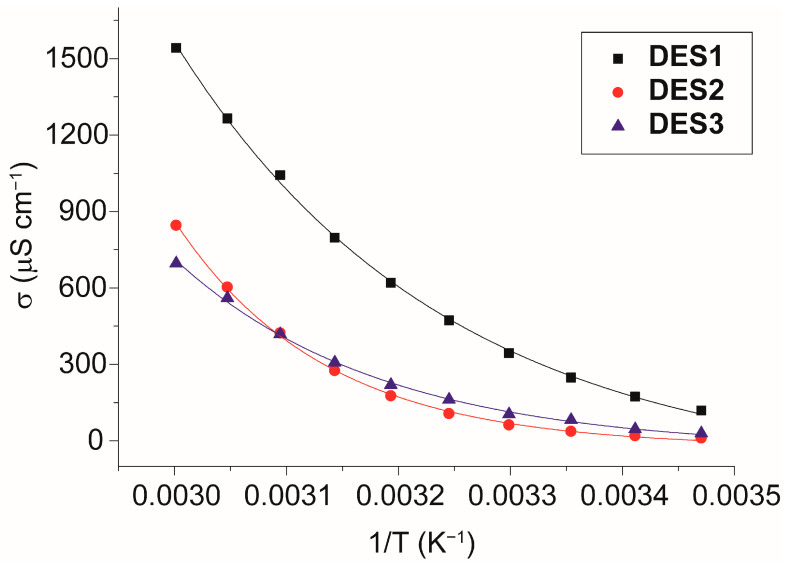
Conductivity of samples measured in the temperature range 288.15–338.15 K. Solid lines represent the best fit equations (*R*^2^ > 0.99 in all the cases).

Another important property of DESs containing salts is the conductivity, as it provides important information about the size and mobility of the ions. Figure 3 shows the conductivity values for DES1–3 in the 288.15–338.15 K range. The values are quite different, denoting remarkable differences in the structure of the three liquids. Such differences can be explained on the basis of their viscosity (see later) or the aggregation of their ions. 

From the exponential trend with 1/T, the activation energies of conductivity can be evaluated and are 34 ± 1, 63 ± 1, and 45 ± 1 kJ mol^−1^ for DES1, DES2, and DES3, respectively (see Table 4). DES2 shows the highest value for *E_a(σ)_*, indicating the lowest mobility at room temperature and the most robust ionic aggregation. This can be due to the peculiar structure of glycolic acid, which possesses highly polar groups on both sides of its chemical structure, possibly leading to head-to-tail hydrogen-bonded aggregates. This is partially confirmed by the fact that at high temperatures, the conductivity becomes higher than in the case of DES3, indicating that temperature destroys, at least partially, ionic aggregates.

### 2.5. Rheological Properties

**Figure 4:**
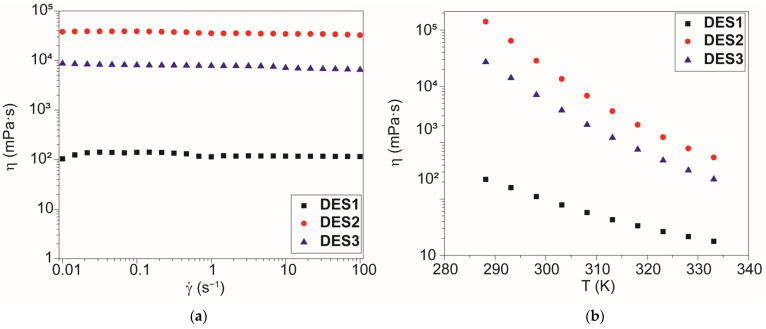
(**a**) Measurement of dynamic viscosity as a function of shear rate for the samples DES1–3. (**b**) Viscosity of samples measured in the temperature range 288.15–333.15 K.

The viscosity of DES1–3 was first measured at 298.15 K as a function of shear rate in order to determine the general flow behavior (Figure 4a). Later, we studied the viscosity dependence on temperature at a fixed shear rate (10 Hz, Figure 4b).

The viscosity (*η*) values obtained follow the same trend as that observed with density (DES2 > DES3 > DES1), and also in this case, the measured values are remarkably different from those of the pure acid precursors (*η* of acetic acid at 298.15 K [47]: 1.115 mPa·s; η of levulinic acid at 308.15 K [48]: 18 mPa·s), suggesting the establishment of interactions among the precursors which significantly decrease the overall system mobility, i.e., the formation of head-to-tail hydrogen-bonded aggregates. It is worth noticing that the high values of DES2 and DES3 hamper their use in many room-temperature applications because the high viscosity of the solvent slows down the mass and heat transfer of any process. In these cases, an increase in the reaction temperature or the addition of a proper amount of cosolvent (which reduces viscosity without breaking the DES structure) may be evaluated. 

The rheological behavior of the samples was mathematically evaluated using the Ostwald–de Waele power-law equation (Equation (7)). The fitting procedure (Figure S2) showed that DES1 had *n* = 0.999 ± 0.001; thus, the power-law flow index can be approximated as 1, and the viscosity can be considered constant in the entire shear rate range, with a Newtonian behavior. On the contrary, the other two samples presented a reduction in viscosity at a higher shear rate, with a power-law flow index of 0.93 ± 0.01 for DES2 and 0.955 ± 0.001 for DES3. This behavior denoted a shear-thinning behavior of these fluids.

In a second assessment, the viscosity at a fixed shear rate (10 Hz) was measured in the range 288.15–333.15 K. The dependence of viscosity on temperature can be described satisfactorily with a simple exponential decay in the entire temperature range (as proposed by the Arrhenius equation, Equation (8)). The data were fitted with the Arrhenius equation in a selected temperature range in order to determine the activation energy of the process (Table 4).

### 2.6. NMR Characterization

**Figure 5:**
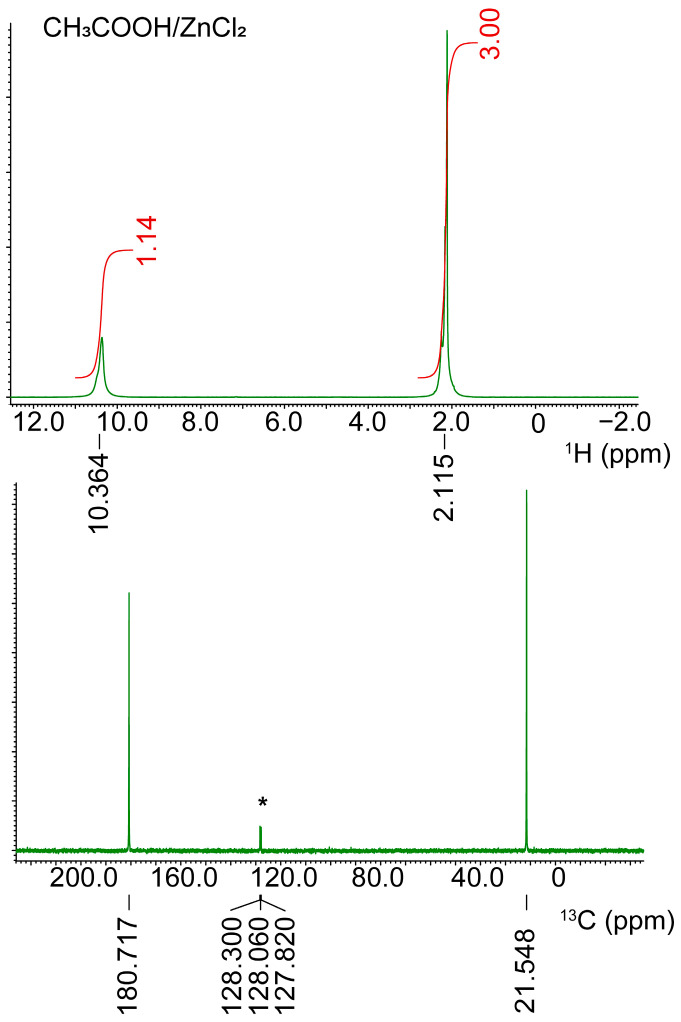
^1^H (up) and ^13^C{^1^H} NMR spectra of pure DES1 **(in green) with the correspondent integrated area (in red)**. A sealed capillary containing deuterated benzene (peak labeled with an asterisk) was used as a reference.

As all the liquids DES1–4 are diamagnetic in nature, they can be characterized by nuclear magnetic resonance (NMR) spectroscopy (Figure 5 and Supporting Information Figures S3–S5). For DES1, the peaks are easily recognizable: the methyl group resonates at 2.115 (protons) and 21.55 ppm (carbon), and the carboxylic moiety resonates at 10.364 (proton) and 180.72 ppm (carbon).

The peak broadening in some cases is severe (e.g., DES2 and DES3, Supporting Information), likely due to the high viscosity of the liquids. In the case of DES1 and DES4, the NMR peaks of DESs can be compared to those of pure liquids, obtained at the same temperature (22.1 °C). For DES1, both peaks of the ^1^H NMR spectrum are shifted downfield: the peak due to methyl protons moves from 2.701 (pure acetic acid) to 2.115 ppm, while that due to the acidic proton shifts from 12.268 to 10.364 ppm. On the contrary, the peaks in the ^13^C{^1^H} move upfield: 20.62 and 178.60 for pure acetic acid, 21.54 and 180.72 ppm for DES1. The same phenomenon is observed when comparing pure formic acid (^1^H NMR spectrum 11.848, 8.930 ppm; ^13^C{^1^H} NMR spectrum 167.50 ppm) and DES4 (^1^H NMR spectrum 9.270, 8.127 ppm; ^13^C{^1^H} NMR spectrum 169.44 ppm).

## 3. Experimental Details

### 3.1. Determination of Water Content

The water content of acetic acid and formic acid was determined using a HI 904 Karl Fischer Coulometric Titration instrument from Hanna Instruments at room temperature. Liquid compounds were directly injected into the instruments and analyzed, while solid ones were dissolved in anhydrous DMSO (water content of DMSO 41.7 ppm) just before the analysis. DMSO was kept over activated molecular sieves (4 Å), and all the procedures were performed with Schlenk techniques and under N_2_. The results for relevant components are the following: ZnCl_2_, 25,400 ppm of water; glycolic acid, 31,800 ppm; acetic acid, 1908 ppm; formic acid, 7066 ppm. Levulinic acid, having a ketone moiety, was not suitable for Karl Fischer analysis, and thermogravimetric analysis (TGA) was used instead to evaluate the water present. The water content of levulinic acid was determined by percent mass loss in isotherm at 80 °C and was found to be 18,000 ppm. As a verification of consistency between the two measurement methodologies, the water content of ZnCl_2_ was also measured by thermogravimetry and was found to be practically coincident with that obtained by Karl Fischer titration.

### 3.2. LBDES Preparation

Different mixtures were prepared by mixing the metal precursor with the desired acid (ZnCl_2_/acetic acid, molar ratio 1:3 = DES1; ZnCl_2_/glycolic acid, molar ratio 1:4 = DES2; ZnCl_2_/levulinic acid, molar ratio 1:3 = DES3; ZnCl_2_/formic acid, molar ratio 1:3 = DES4) in suitable quantities under mild heating (maximum 100 °C for some hours) until a homogeneous clear (colorless or pale yellow) liquid appeared. Then, the liquid was allowed to rest for at least 24 h at room temperature in order to check its stability. In particular, we detected the absence of aggregation phenomena in the samples, which were still homogeneous liquid mixtures.

### 3.3. Experimental and Theoretical Solid–Liquid Phase Diagram

The stable liquids were identified as DESs by comparing the experimental solid–liquid phase diagrams with the theoretical ones by measuring the melting points at different molar fractions [49]. The samples were put in a Dewar flask with a CO_2_/acetone mixture, and the melting points were measured with a thermometer (±0.1 °C). The melting points were taken in triplicate to avoid a kinetic effect on the melting of the mixtures, and the values showed standard deviation values < 2 °C. More sophisticated methods, such as differential scanning calorimetry, were unfeasible due to the corrosive nature of the LBDESs toward the sample holder.

The solid–liquid theoretical curves were determined by using Equation (1), which represents the solid–liquid equilibrium curve:

(1) $$\ln\left({x_{i}\gamma }_{i}\right)=\frac{{∆}_{m}H_{i}}{R}\cdot \left(\frac{1}{T_{m,i}}-\frac{1}{T}\right)+\frac{{∆}_{m}C_{p,\;i}}{R}\;\cdot \;\left[\frac{T_{m,i}}{T}-ln\left(\frac{T_{m,i}}{T}\right)-1\right]$$

where *x_i_* is the mole fraction of component *i*, *γ_i_* is its activity coefficient in the liquid phase, Δ*_m_H_i_* and *T_m,i_* are its melting enthalpy and temperature, respectively, Δ*_m_C_p,i_* is its heat capacity change upon melting, *R* is the ideal gas constant, and *T* is the absolute temperature of the system. Equation (1) was previously used by many authors to describe the solubility curves of many solid compounds [50], and more specifically to describe the phase diagram of various DESs [13]. It can be simplified by considering the effect of the temperature on the enthalpies of melting negligible, that is, neglecting the second term depending on the Δ*_m_C_p,i_*, thus obtaining Equation (2):

(2) $$\ln\left(x_{i}\gamma_{i}\right)=\frac{{∆}_{m}H_{i}}{R}\cdot \left(\frac{1}{T_{m,i}}-\frac{1}{T}\right)$$

This approximation is commonly accepted for the study of similar systems. The theoretical melting temperatures were determined from the theoretical curves by considering the activity coefficients *γ_i_* = 1. The experimental *γ_i_* values were determined via Equation (3) by using the experimentally observed melting temperatures:

(3) $$\gamma_{i}=\frac{exp\left[\frac{{∆}_{m}H_{i}}{R}\left(\frac{1}{T_{m,i}}-\frac{1}{T}\right)\right]}{x_{i}}$$

The literature data presented in Table 5 were used for Equations (2) and (3).

### 3.4. Density 

Density (ρ) measurements were performed using an Anton Paar DMA 55 Densimeter, with a water-circulating thermostat to control the temperature with a precision of ±0.01 K. The densimeter was calibrated with air and degassed water at all the measurement temperatures. The measurements were performed every 5 K in the range 293.15–338.15 K. The error related to the technique uncertainty was 2 ± 10^−5^ g·cm^−3^, while the error in the measurement (calculated by repeating the measurements on three samples of DES1 prepared independently at representative temperatures) was about ±10^−3^ g·cm^−3^, due to the inherent variability resulting from the sample preparation procedure. Then, the density data as a function of temperature were fitted with a linear regression through the following equation:

*ρ* = *b* + *aT* (4)

where *ρ* is the density (g·cm^−3^), *T* is the temperature (K), and *a* (g·cm^−3^·K^−1^) and *b* (g·cm^−3^) are the fitting parameters. All the data were fitted satisfactorily with R^2^ > 0.9999. The parameters *a* and *b* obtained from the fitting procedure were used to calculate the isobaric expansibility coefficient (*α_p_*) at 298.15 K through the following equation:

(5) $$\alpha_{p}=\frac{1}{V}{\left(\frac{\partial V}{\partial T}\right)}_{p}=-\frac{1}{\rho }\;{\left(\frac{\partial P}{\partial T}\right)}_{p}=\frac{a}{\rho \left(T\right)}$$

where *V* is the volume (cm^3^), *T* is the temperature (K), and *ρ* is the density (g·cm^−3^). 

### 3.5. Electrical Conductivity

Electrical conductivity (*σ*) was measured with an Analytical Control conductometer (model 120) equipped with a platinum cell (cell constant = 1.05 cm^−1^) in the temperature range 288.15–333.15 K (with a temperature step of 5 K), maintaining a constant sample temperature with a glass-jacketed beaker connected to a thermostatic bath. Each value was recorded after 20–25 min of homogenization for each temperature selected. The conductivity dependence on temperature was described with the Arrhenius equation (Equation (6)) in an optimized temperature range.

(6) $$\sigma =A_{\sigma }\;e^{\frac{{-E}_{a(\sigma )}}{R\;T}}$$

where *σ* is the specifical electrical conductivity (S·cm^−1^), *A_σ_* and *E_a(σ)_* are the pre-exponential factor (S·cm^−1^) and the activation energy related to conductivity (J·mol^−1^), respectively, *R* is the gas constant (8.3145 J·mol^−1^·K^−1^), and *T* is the temperature (K). To ensure consistency between results, the same sample was used for conductivity and viscosity experiments. The error related to technique uncertainty was ±1 μS·cm^−1^.

### 3.6. Viscosity

Viscosity (*η*) measurements were performed with a Rheo Stress rheometer by Thermo Scientific using a plate/plate arrangement with a radius of 60 mm in TEFLON-coated stainless steel, due to the corrosive nature of the samples analyzed. Flow curves were measured in the range of shear rates between 0.01 and 1000 Hz (for DES1) or between 0.01 and 147 Hz for DES2–3 at a fixed temperature (298.15 K). Each point was taken as the integral of the signal measured for 10 s after reaching the steady state. The rheological behavior of the samples was determined by applying the Ostwald–de Waele power-law equation [52] to the graph of shear stress vs. shear rate (Equation (7)):

(7) $$\tau =k\;{\dot{\gamma }}^{n}$$

where *τ* is the shear stress (mPa), *k* (mPa·s) is a constant, *n* (dimensionless) is the power-law flow index, $\dot{\gamma }$ is the shear rate (Hz). Newtonian fluids have *n* = 1, while shear-thinning (pseudoplastic) or shear-thickening (dilatant) fluids have *n* < 1 or *n* > 1, respectively.

The viscosity dependence on temperature was measured between 288.15 and 333.15 K (with a temperature step of 5 K) at a fixed shear rate (10 Hz). The viscosity dependence on temperature was described with the Arrhenius equation in a temperature range opportunely chosen (Equation (8)):

(8) $$\eta =A_{\eta }\;e^{\frac{E_{a(\eta )}}{R\;T}}$$

where *η* is the dynamic viscosity (Pa·s), *A_η_* and *E_a(η)_* are the pre-exponential factor (Pa·s) and the activation energy (J·mol^−1^) related to viscosity (*η*), respectively, *R is* the gas constant (8.3145 J·mol^−1^·K^−1^), and *T* is the temperature (K).

The temperature was controlled using a Peltier module system. Error in the measurements (calculated as the standard deviation of three samples of DES1 prepared independently) was about ±50 mPa·s, due to the inherent variability resulting from the sample preparation procedure.

### 3.7. NMR

^1^H and ^13^C NMR measurements were carried out on an FT-NMR Joel JNM-ECZ500R MHz with an HFX probe at the indicated temperatures. The temperature of the instrument was calibrated using a sample of pure ethylene glycol and using Equation (9):

(9) $$T=\left(4.637-\Delta \right)/0.009967$$

where Δ is the chemical shift separation between the two peaks of ethylene glycol. In all the cases in which a pure liquid was studied, a sealed capillary containing benzene-d6, DMSO-d6, or acetone-d6 was used for locking and to allow the necessary shimming procedure.

## 4. Conclusions

In conclusion, four different LBDESs have been characterized, in view of future, potential applications. Among the diamagnetic metal chlorides studied here, only zinc(II) chloride showed some propensity for the formation of stable Lewis/Brønsted DESs. In the case of ZnCl_2_/formic acid, the LBDES was stable at room temperature but unstable under heating, releasing carbon monoxide, and it has been discarded for safety. The other three systems, ZnCl_2_/acetic acid, ZnCl_2_/glycolic acid, and ZnCl_2_/levulinic acid, were stable and safe to use.

In particular, their nature as deep eutectic solvents has been demonstrated by comparing ideal and experimental melting points, and they have been characterized by means of density, conductivity, and viscosity in the range of 15–60 °C. The Brønsted acid very effectively tuned all the properties of the liquid, giving a wide range of choices for potential applications. In the case of glycolic acid, experimental data suggest that head-to-tail aggregates can be present in the solution.

The combined Lewis and Brønsted acidity of these DESs is potentially useful for many acid- or zinc-catalyzed reactions, with the added benefit that NMR techniques can be used for kinetic or in-depth characterization studies.

## Figures and Tables

**Table 1:** Successful (✓) and unsuccessful (✗) combinations for the preparation of diamagnetic acidic DESs. In parentheses, the corresponding molar ratio (acid:metal salt) is indicated.

Acid	+ZrOCl_2_·8H_2_O	+ZnCl_2_	+SnCl_2_	+AlCl_3_·6H_2_O
Acetic acid	✗	✓ (3:1, DES1)	✗	✗
Glycolic acid	✗	✓ (4:1, DES2)	✓ (6:1) ^a^	✗
Oxalic acid	✗	✗	✗	✗
MSA ^b^	✓ (7:1) ^a^	✗	✗	✗
*p*TSA ^c^	✗	✗	✗	✗
Levulinic acid	✗	✓ (3:1, DES3)	✗	✗
Formic acid	✓ (2.5:1) ^a^	✓ (3:1, DES4)	✗	✗

^a^ unstable liquids; ^b^ MSA = methanesulfonic acid; ^c^ *p*TSA = para-toluenesulfonic acid.

**Table 2:** Activity coefficients for DES1–4 ^a^.

Sample	*γ_Brønsted acid_*	*γ*_ZnCl2_
DES1	0.31	0.12
DES2	0.18	0.21
DES3	0.62	0.20
DES4	0.36	0.13

^a^ Calculated according to Equation (3), at the melting temperature observed experimentally.

**Table 3:** Parameters describing the density dependence on temperature for DES1–3.

Parameter ^a^	DES1	DES2	DES3
*a* (fitting) (g·cm^−3^ K^−1^)	−0.001200	−0.000961	−0.000926
*b* (fitting) (g·cm^−3^)	1.86959	1.95845	1.68288
*α_P_* (10^4^) (K^−1^) ^b^	7.94	5.75	6.58

^a^ *a* and *b* are the slope and the intercept of the fitting line, as expressed in Equation (4). ^b^ Calculated at 298.15 K.

**Table 4:** Activation energies of DES1–3 calculated by Arrhenius equation for both viscosity and conductivity.

	*E_a(η)_* (kJ·mol^−1^)	*E_a(σ)_* (kJ·mol^−1^)
DES1	52 ± 1	34 ± 1
DES2	113 ± 0.9	63 ± 1
DES3	95.4 ± 0.1	45 ± 1

**Table 5:** Melting point and enthalpy of fusion values for the substances used in this work. Data from reference [51].

Substance	*T_m_* (K)	Δ*_m_H* (kJ·mol^−1^)
Acetic acid	289.75	11.72
ZnCl_2_	563.15	7.322
Glycolic acid	348.15	17.30
Levulinic acid	306.15	9.22
Formic acid	281.45	12.68

## Data Availability

Data are contained within the article and Supplementary Materials.

## Supplementary Materials

The following supporting information can be downloaded at https://www.mdpi.com/article/10.3390/molecules28248054/s1: Figure S1: A glass tube containing I_2_O_5_ after contact with the gas produced by DES4 (ZnCl_2_/formic acid). The purple phase (I_2_), due to CO oxidation, can be easily appreciated; Table S1: Density measurements for DES1–3; Table S2: Conductivity measurements for DES1–3; Table S3: Viscosity measurements for DES1–3; Figure S2: Fitting via power-law equation of shear stress vs. shear rate of DES1–3; Figure S3: ^1^H (up) and ^13^C{^1^H} NMR spectra of pure DES2. A sealed capillary containing deuterated benzene (peak labeled with an asterisk) was used as reference; Figure S4: ^1^H (up) and ^13^C{^1^H} NMR spectra of pure DES3. A sealed capillary containing deuterated benzene (peak labeled with an asterisk) was used as reference; Figure S5: ^1^H (up) and ^13^C{^1^H} NMR spectra of pure DES3. A sealed capillary containing deuterated benzene (peak labeled with an asterisk) was used as reference.
