# Supplementary material for: Preparation and Characterization of Zinc(II)-Based Lewis/Brønsted Acidic Deep Eutectic Solvents
Source: Molecules. 2023 Dec 12;28(24):8054. doi: 10.3390/molecules28248054 (PMC10745514; doi:10.3390/molecules28248054)
Supplement: Supplementary file 1 [file molecules-28-08054-s001.zip › molecules-2733809-supplementary.pdf]

SUPPORTING INFORMATION

Preparation and characterization of zinc(II)-based Lewis/Brønsted acidic deep eutectic solvents

Chiara Pelosi,[a] Aldo Quaranta,[a] Marco Rollo,[a] Elisa Martinelli[a], Celia Duce[a,b] Gianluca Ciancaleoni,[a,*] Luca Bernazzani[a,*]

[a] Department of Chemistry and Industrial Chemistry, University of Pisa, via Giuseppe Moruzzi 13, I-56124 Pisa, Italy.

[b] Istituto nazionale di ottica (INO-CNR) area di Pisa, Centro Nazionale delle Ricerche, Pisa, Via Moruzzi, 56124, Pisa (PI), Italy.

Corresponding authors: gianluca.ciancaleoni@unipi.it; luca.bernazzani@unipi.it

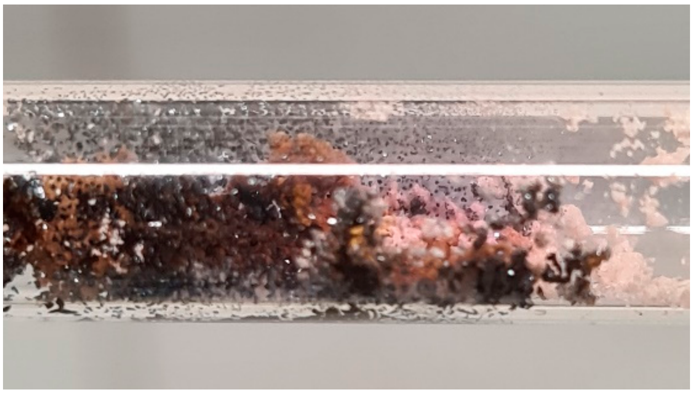

Figure S1. A glass tube containing I_2O_5 after the contact with the gas evolved by **DES4** ($ZnCl_2$/formic acid). The purple phase (I_2), due to CO oxidation, can be easily appreciated.

Table S1. Density measurements for **DES1-3**.

**T(°C)**	**ϱ (g·cm^{-3})**		
	**DES1**	**DES2**	**DES3**
15	1.525	1.682	1.416
20	1.519	1.677	1.411
25	1.513	1.672	1.407
30	1.507	1.667	1.402
35	1.501	1.662	1.398
40	1.495	1.658	1.393
45	1.489	1.653	1.388
50	1.483	1.648	1.384
55	1.477	1.643	1.379
60	1.471	1.638	1.374

Table S2. Conductivity measurements for **DES1-3**.

**T(°C)**	**σ (μS·cm^{-1})**		
	**DES1**	**DES2**	**DES3**
15	118	10	29
20	173	19	46
25	248	37	82
30	344	62	104
35	472	106	162
40	620	176	220
45	797	275	308
50	1043	423	419
55	1265	603	560
60	1542	846	697

Table S3. Viscosity measurements for **DES1-3**.

**$\dot{\gamma}$ (s^{-1})**	**η (mPa·s)[a]**		
	**DES1**	**DES2**	**DES3**
0.010	104	37930	8736
0.015	124	38417	8573
0.022	138	38699	8373
0.032	141	38690	8308
0.046	139	38806	8219
0.068	137	38787	8168
0.100	140	38644	8094
0.147	141	38361	8045
0.215	139	38044	8005
0.316	135	37616	7972
0.464	130	37175	7948
0.681	117	35791	7888
1.000	114	35380	7831
1.468	120	35227	7788
2.154	118	35371	7781
3.162	119	35403	7739
4.641	118	34881	7694
6.812	118	34672	7568
9.999	117	34309	7111
14.676	117	34434	6955
21.542	117	34313	6854
31.620	117	34188	6795
46.410	116	33734	6732
68.121	116	33358	6637
99.987	115	32509	6511
146.763	115	30764	6370
215.416	115		
316.192	115		
464.105	116		
681.217	116		
999.881	115		

T(°C)	η (mPa·s)[a]		
	DES1	DES2	DES3
15	222	140600	27112
20	159	64002	14091
25	110	28365	7041
30	78	13475	3747
35	58	6808	2083
40	43	3618	1222
45	33	2068	755
50	26	1247	485
55	22	784	322
60	18	545	224

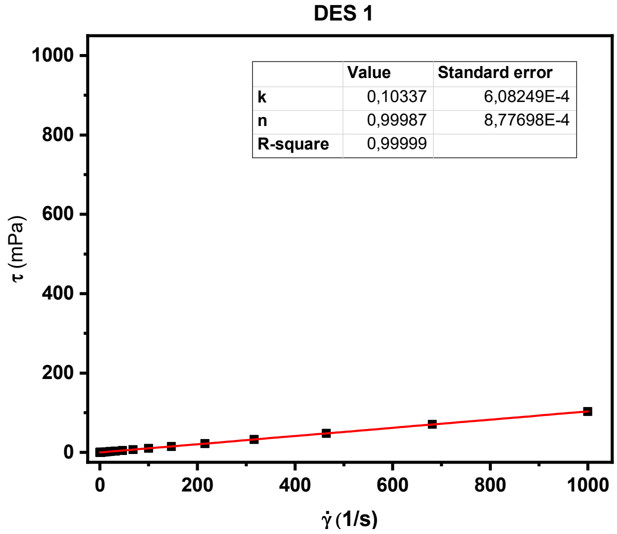

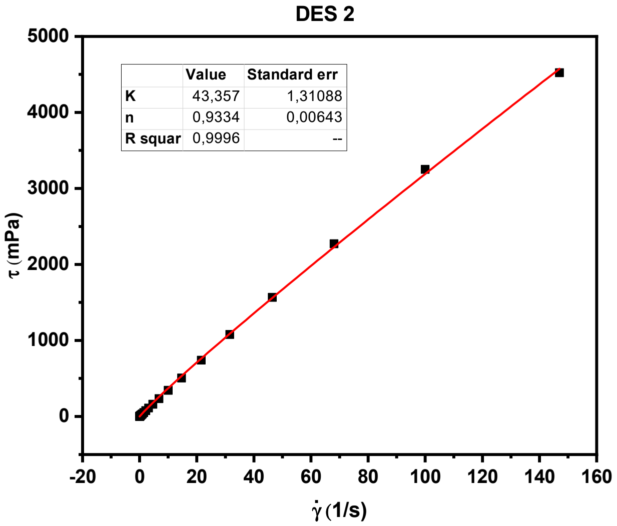

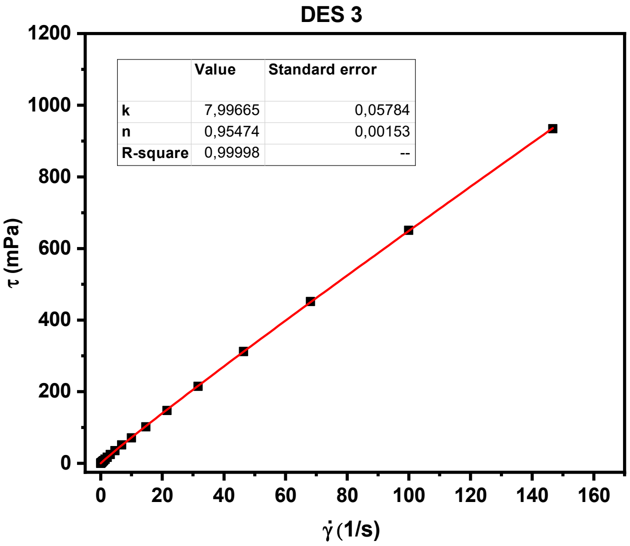

Figure S2. Fitting via power-law equation of shear stress vs shear rate of **DES1-3**.

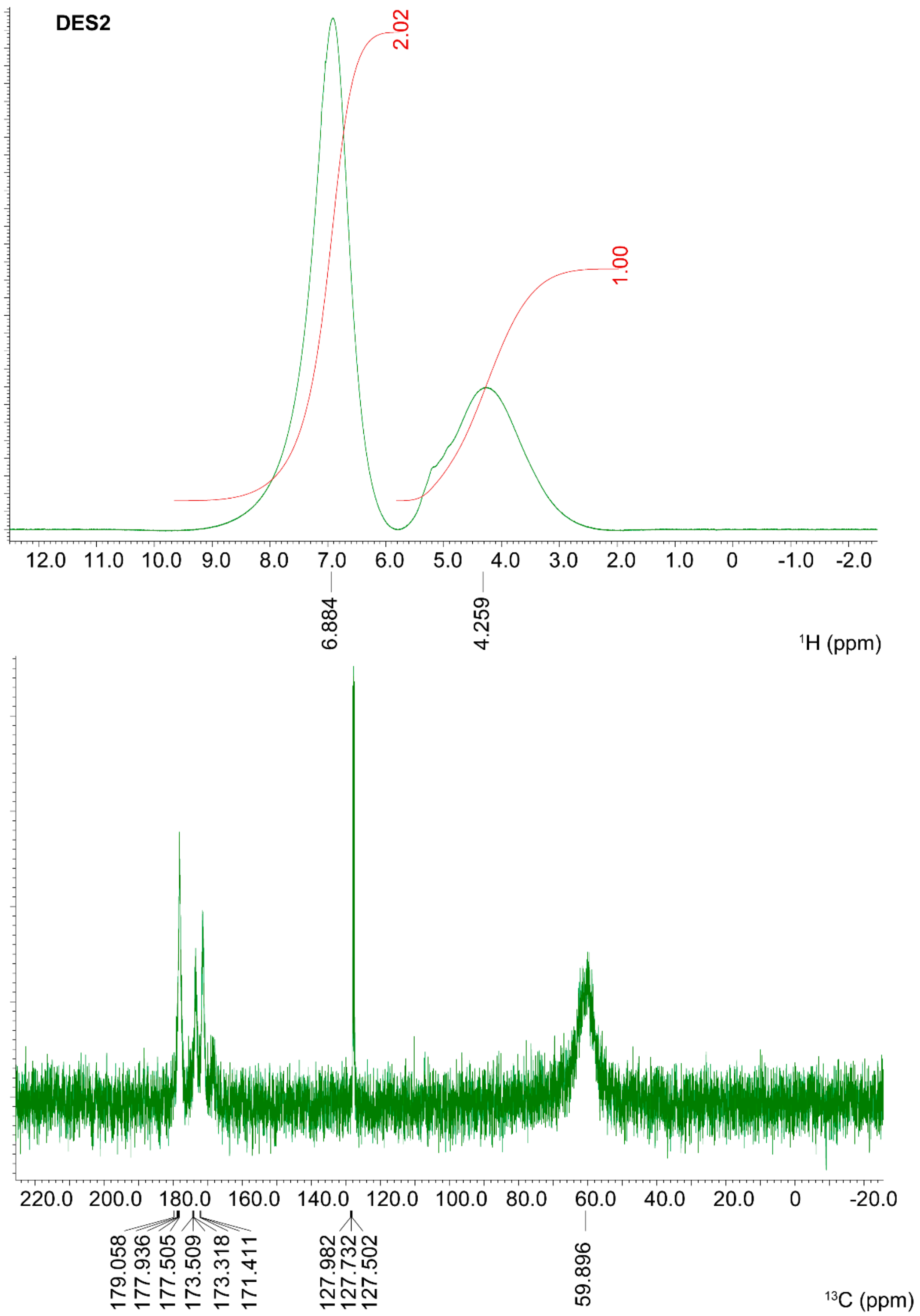

Figure S3. ^{1}H (up) and $^{13}C\{^{1}H\}$ NMR spectra of pure **DES2**. A sealed capillary containing deuterated benzene (peak labelled with an asterisk) has been used as reference.

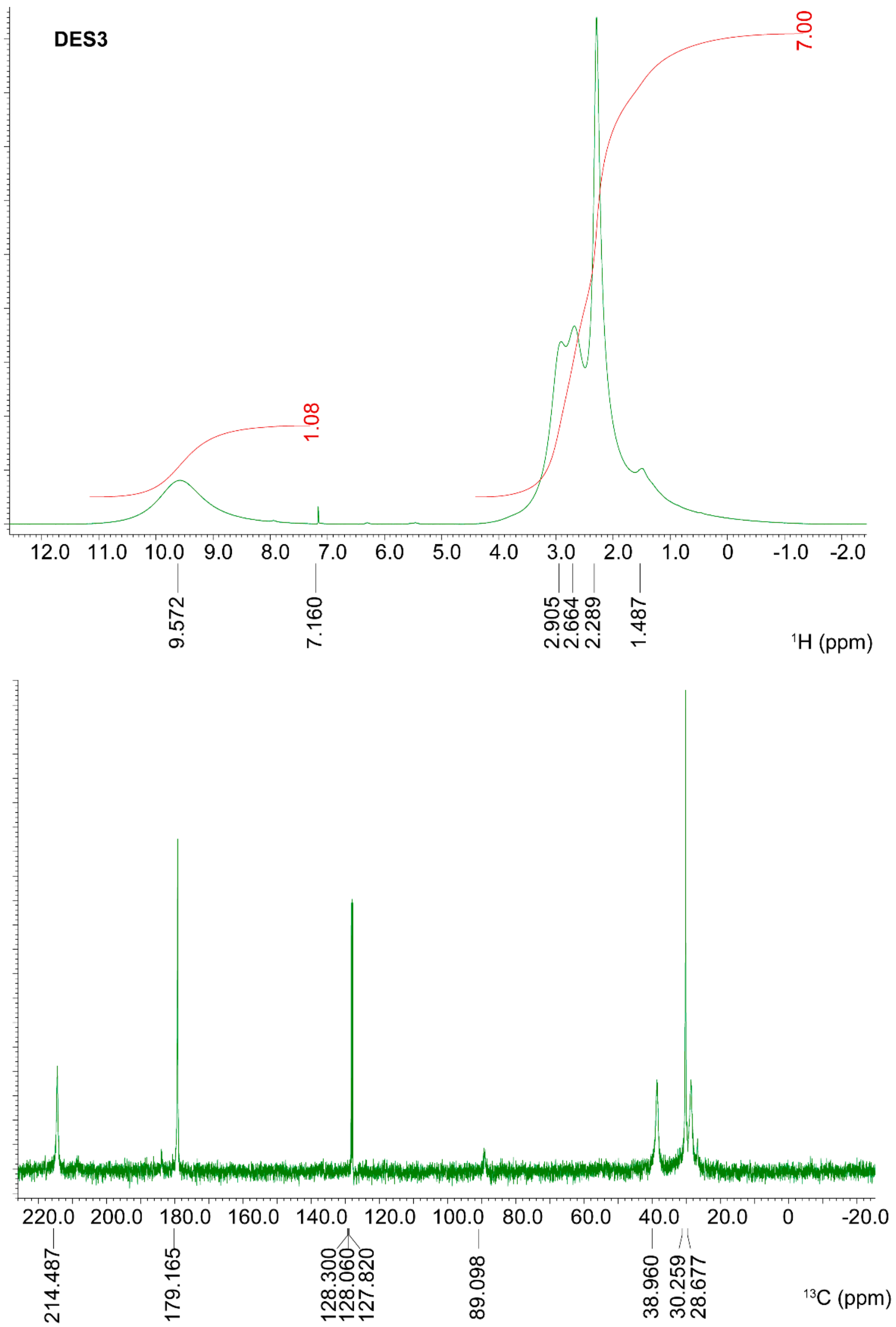

Figure S4. ^{1}H (up) and $^{13}C\{^{1}H\}$ NMR spectra of pure **DES3**. A sealed capillary containing deuterated benzene (peak labelled with an asterisk) has been used as reference.

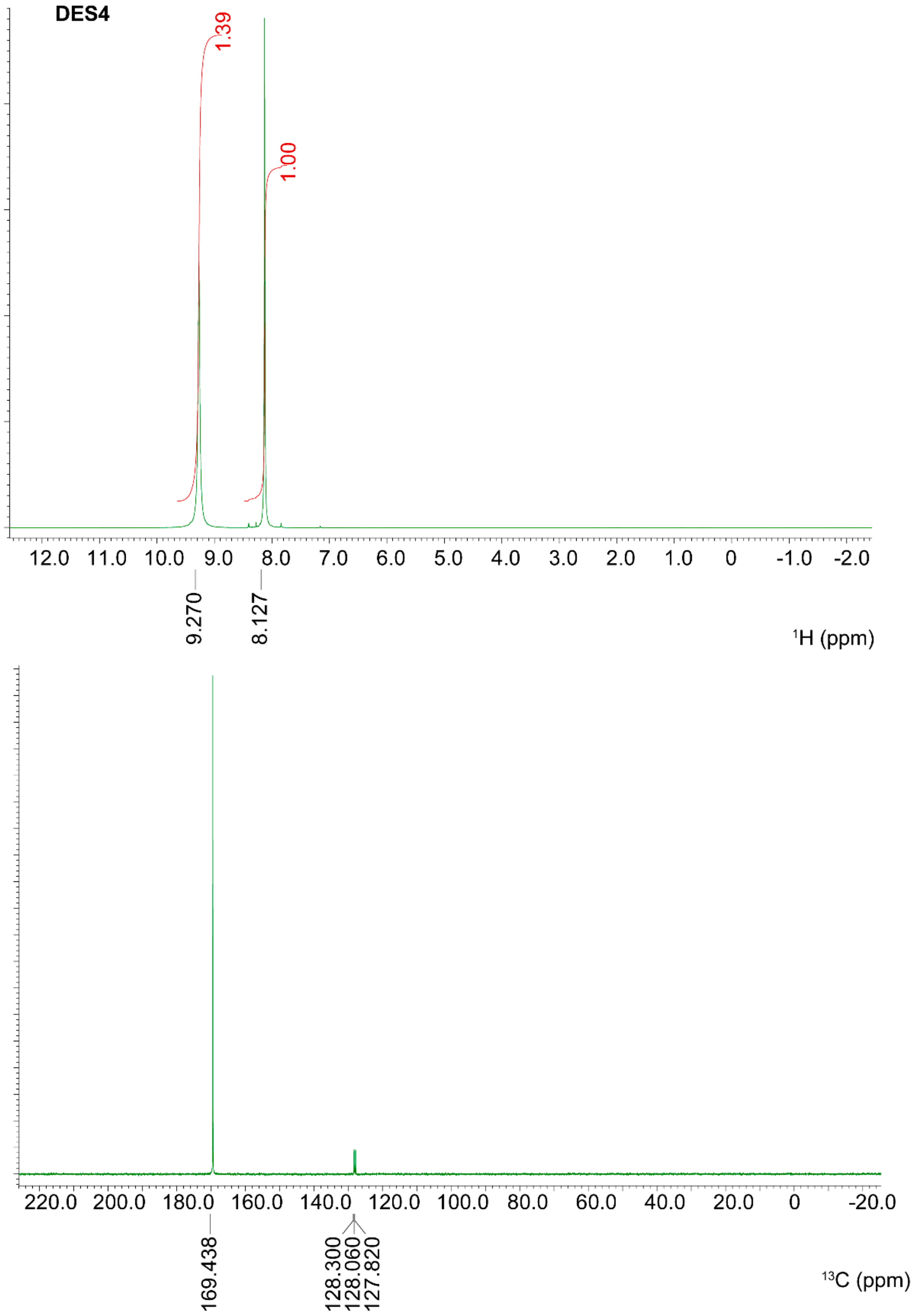

Figure S5. ^{1}H (up) and $^{13}C\{^{1}H\}$ NMR spectra of pure **DES3**. A sealed capillary containing deuterated benzene (peak labelled with an asterisk) has been used as reference.
